# Regulatory effect of *Ganoderma lucidum* and its active components on gut flora in diseases

**DOI:** 10.3389/fmicb.2024.1362479

**Published:** 2024-03-18

**Authors:** Xinjie Qin, Zinan Fang, Jinkang Zhang, Wenbo Zhao, Ni Zheng, Xiaoe Wang

**Affiliations:** ^1^College of Food Engineering, Jilin Engineering Normal University, Changchun, China; ^2^Jilin Province Key Field of Social Sciences (Food Industry) Research Base, Changchun, China

**Keywords:** *Ganoderma lucidum*, active ingredients, gut flora, medicinal value, regulation

## Abstract

Driven by the good developmental potential and favorable environment at this stage, *Ganoderma lucidum* is recognized as a precious large fungus with medicinal and nutritional health care values. Among them, polysaccharides, triterpenoids, oligosaccharides, trace elements, etc. are important bioactive components in *G. lucidum*. These bioactive components will have an impact on gut flora, thus alleviating diseases such as hyperglycemia, hyperlipidemia and obesity caused by gut flora disorder. While numerous studies have demonstrated the ability of *G. lucidum* and its active components to regulate gut flora, a systematic review of this mechanism is currently lacking. The purpose of this paper is to summarize the regulatory effects of *G. lucidum* and its active components on gut flora in cardiovascular, gastrointestinal and renal metabolic diseases, and summarize the research progress of *G. lucidum* active components in improving related diseases by regulating gut flora. Additionally, review delves into the principle by which *G. lucidum* and its active components can treat or assist treat diseases by regulating gut flora. The research progress of *G. lucidum* in intestinal tract and its potential in medicine, health food and clinical application were fully explored for researchers.

## 1 Introduction

**Figure 1:**
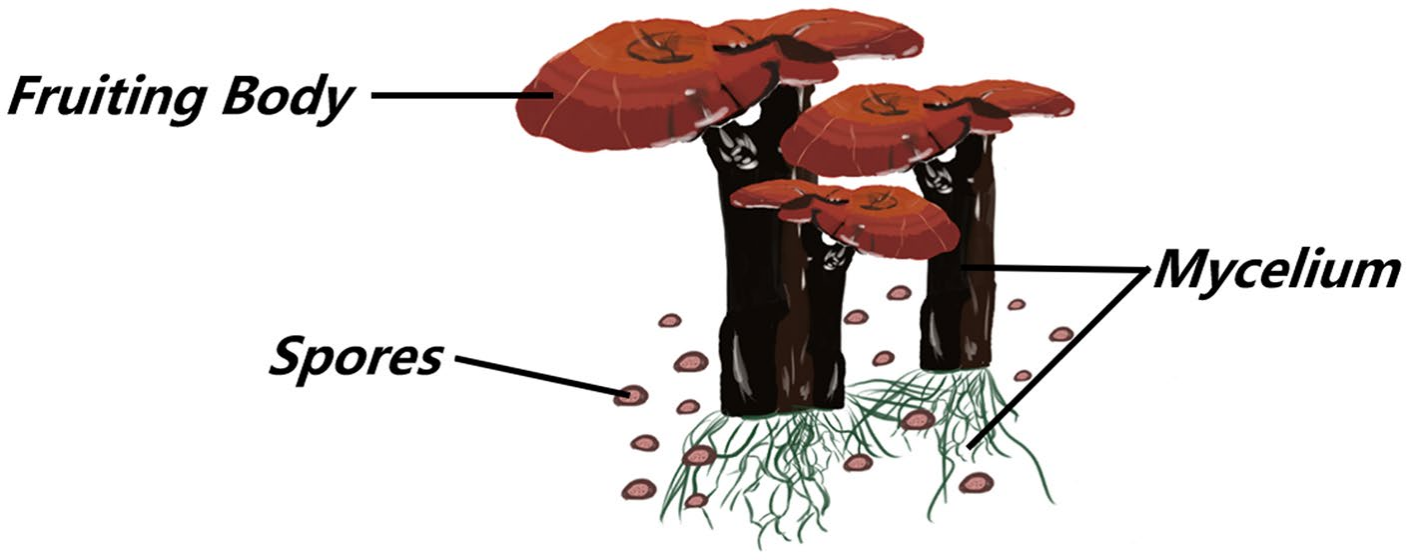
Morphological characteristic map of Ganoderma lucidum.

**Figure 2:**
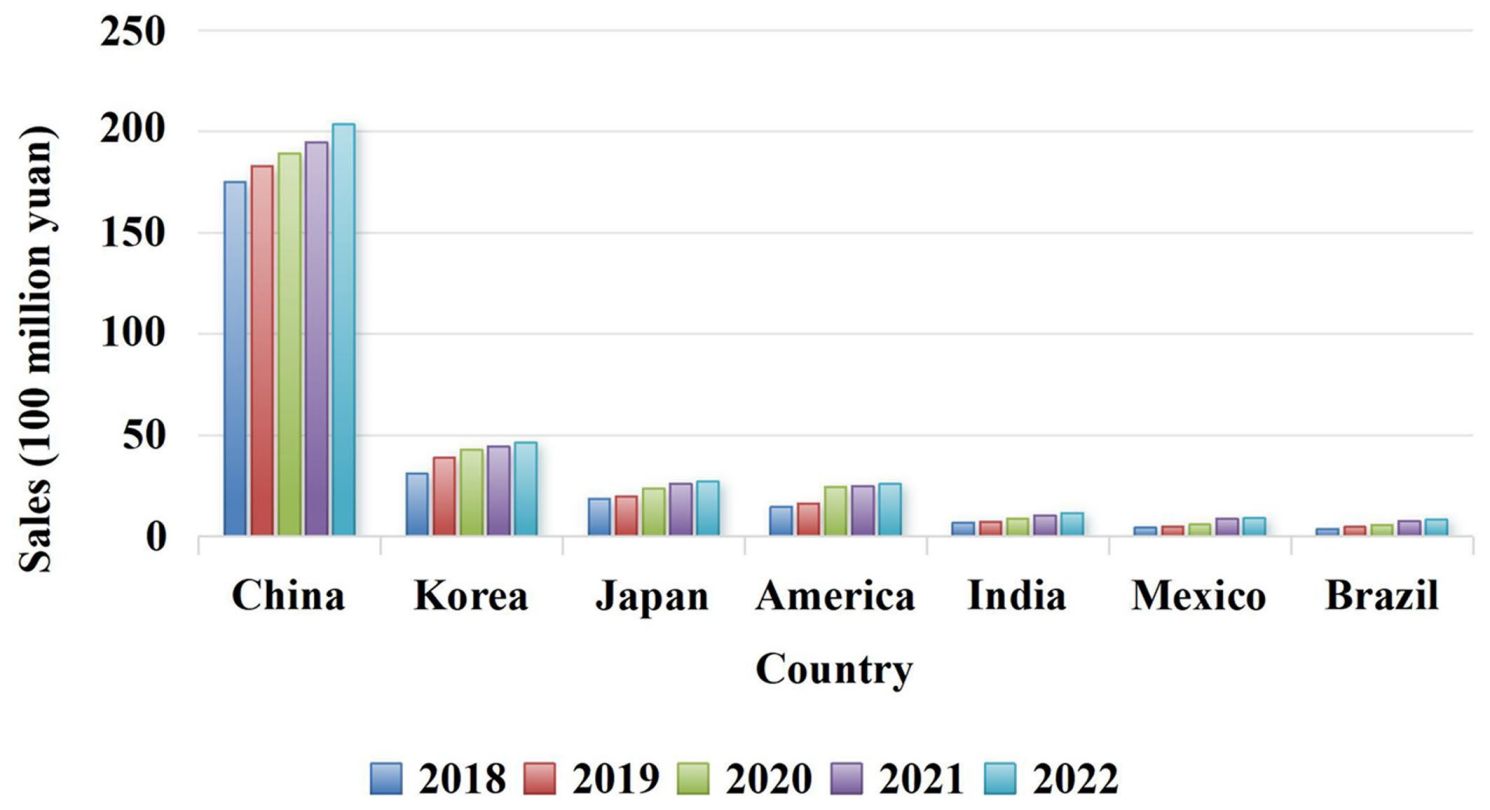
Sales data of *Ganoderma lucidum* in the world.

*Ganoderma lucidum* is a kind of dicotyledonous fungus belonging to the *Ganoderma* genus of the Ganodermataceae family Polyporales order, and Basidiomycota division, which is widely cultivated in China (Blundell et al., 2023). The beneficial characteristics of *G. lucidum* are attributed to bioactive compounds found at two stages of its life cycle: the fruiting body and mycelium. The mycelial stage is the most vigorous phase of the *G. lucidum* life cycle, and spore powder is released for propagation when *G. lucidum* grows and matures (Nakagawa et al., 2018; Figures 1, 2).

According to modern scientific research, *G. lucidum* is composed of 90% water and the remaining 10–40% macromolecules, 3–28% fat and 2% others (fibrous substances and crude proteins). *G. lucidum* also contains vitamins, minerals, metals, inorganic substances, essential fatty acids, amino acids, and other ashes. *G. lucidum* has a variety of secondary metabolites, such as polysaccharides, triterpenoids, adenosine, sterols, alkaloids, oligosaccharides and so on, which are all effective active components (Longgang et al., 2020; Lepakshi et al., 2023). (The chemical structure of the reviewed components is shown in Table 1). A large number of modern pharmacological studies have shown that *G. lucidum* is a mushroom with dual functions as a medicine and food, and possesses biological functions such as anticancer (Jin et al., 2016), anti-inflammatory, antitumor, antiviral, anti-infection, anti-oxidative, immunomodulating, nerve-calming, hepatoprotective, liver-detoxifying, antihypertensive, and antidiabetic activities, as well as the prevention and treatment of cardiovascular diseases (Chen et al., 2016; Yiqiong et al., 2020). In recent years, an increasing number of studies have emphasized the health care function of *G. lucidum* in diseases (Batra et al., 2013), and many researchers have found that the gut flora is closely involved in the biological functions of the secondary metabolites of *G. lucidum*. This review aims to emphasize the pharmacological (Thuy et al., 2023) effects of *G. lucidum* and explore the regulatory mechanism of its active components on gut flora in disease treatment (summarized in Table 2). Through this review, we hope that investigators can better understand the progress of research on *G. lucidum* and its active components in the intestinal field.

**Table 1:** Chemical formula and structure of active components of *Ganoderma lucidum* for regulating gut flora.

Ingredient	Chemical formula	Structure	References
GLP^a^	C_48_H_85_O_36_	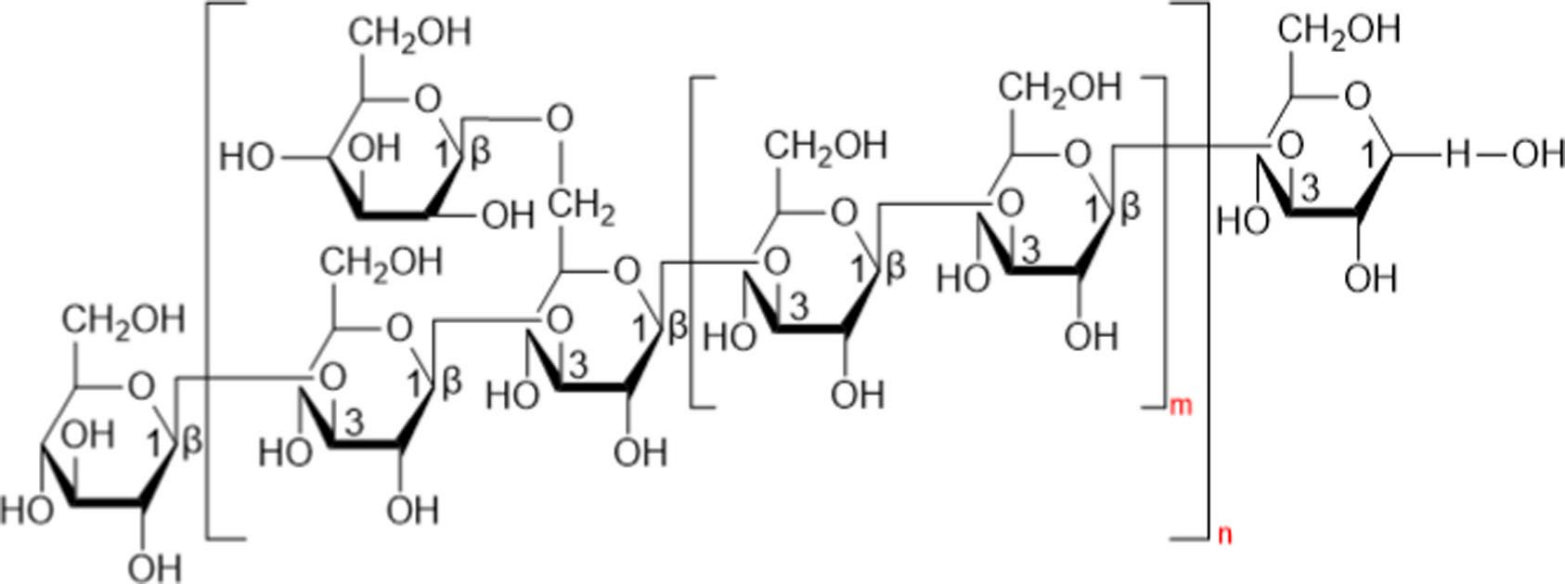	Xu (2010)
GA	C_30_H_42_O_7_	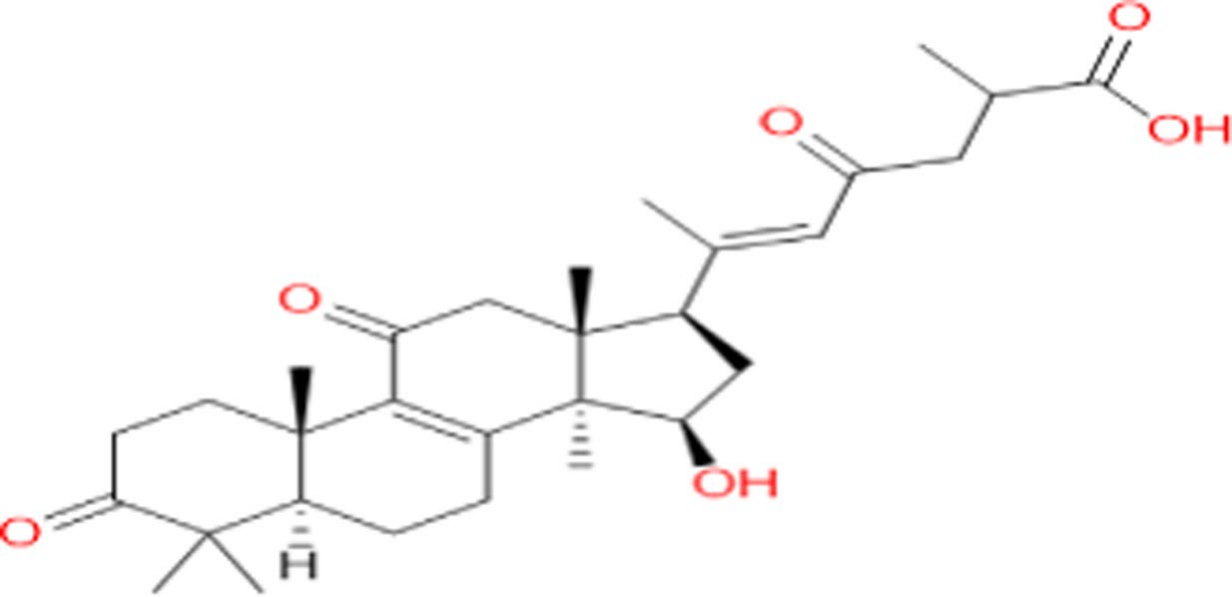	He et al. (2023)
GLO	→6)-β-D-Glcp-(1→, →4)-α-D-Glcp-(1→, α-D-Manp-(1→	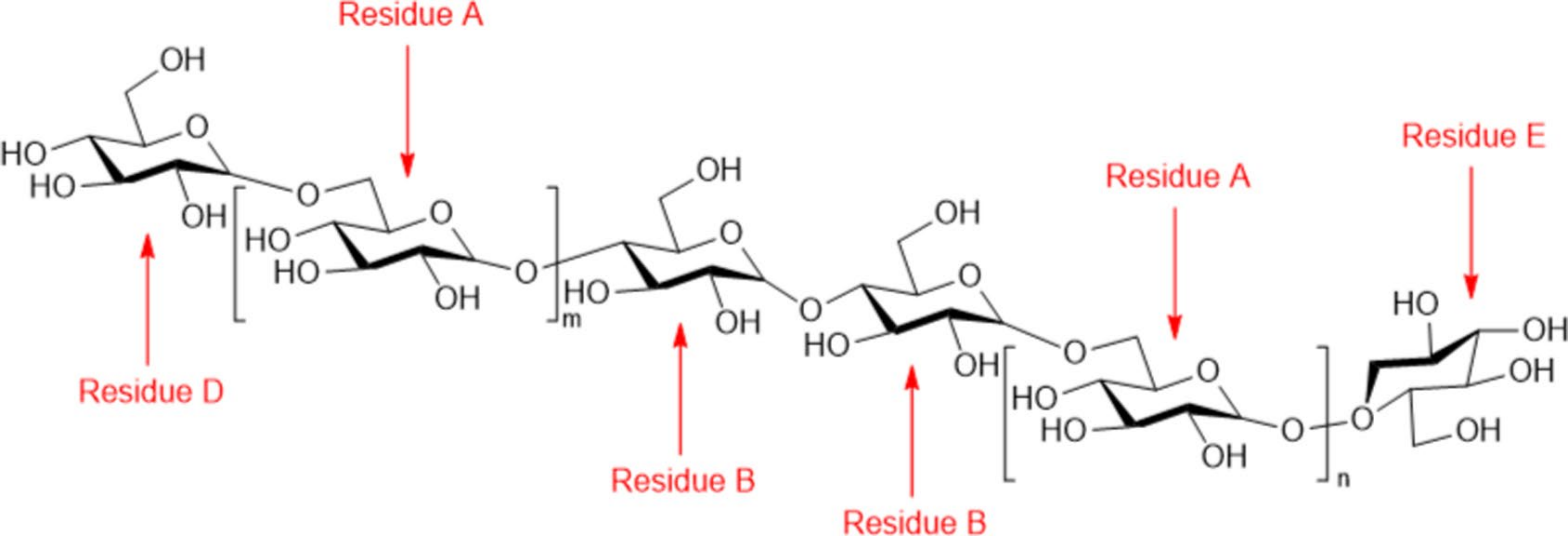	Xia et al. (2022)

^a^GLP, *G. lucidum* polysaccharide; GA, ganoderic acid; GLO, *G. lucidum* oligosaccharide.

**Table 2:** Pharmacological action and main active components of *G. lucidum* and changes of intestinal microbiota.

Main active ingredients	Treat diseases	Animal experiment	Floral change	References
GLP	Regulating intestinal mucosal barrier	SD rats	Ratio of Firmicutes to *Bacteroides* ↓	Jin et al. (2017)
GLPs3	CP	ICR mice	Relative abundance of probiotics and Firmicutes, such as *Lactobacillus*, Lactobacillales, *Roseburia*, and Lachnospiraceae ↑ Relative abundance of *Bacteroides* ↓	Li et al. (2016)
GLP	UC	Wistar rats	The number of *Ruminococcus* 1 ↑ The number of Escherichia-Shigella ↓	Xie et al. (2019)
GLPs	CRC (Prevent colon shortening)	BALB/c mice	The relative abundance (RA) of cecal *Oscillospira* ↓ Find an unknown genus of *Desulfovibrionaceae*	Luo et al. (2018)
GLP	CRC	C57BL/6 mice	The relative abundance of some flora positively related to CRC carcinogenesis (such as Oscillibacter, Desulfovibrio, Alistipes, Lachno-clostridium and Parasutterella) and the relative abundance of Lactobacillus-reuteri and Bifidobacterium-pseudolongum ↓	Guo et al. (2021)
PSG	Liver damage	SD rats	Relative abundance of Ruminococcus, Lactobacillus ↑ Relative abundance of *Prevotella* ↓	Jiang et al. (2021a)
GLSP	ALD	C57BL/6J mice	The relative abundance of beneficial bacteria such as *Bacteroidetes* ↑ The levels of Verrucomicrobiota, *Escherichia*, *Shigella* and harmful bacteria such as Proteobacteria and *Candidatus*-*Saccharibacteria* ↓	Leng et al. (2023)
GL-M	Obesity	C57BL/6J mice	Lactobacillus ↑	Imran et al. (2018)
GL-F			Lactobacillus, Bifidobacterium and Roseburia ↑	
WEGL	Obesity and low-grade chronic inflammation	C57BL/6J mice	The level of *Firmicutes*-to-*Bacteroidetes* ratios and endotoxin bearing *Proteobacteria* ↓	Chang et al. (2015)
GLPP	Aggravated hyperlipidemia and hypercholesterolemia	Wistar rats	Serum and liver lipid profiles were negatively correlated with Jeotgalicoccus, Ignavigranum, Sporosarcina, Bacteroides, Anaerovorax, Parasutterella, Alistipes and Alloprevotella, but positively correlated with Allobaculum, Phascolarctobacterium, Psychrobacter, Enterorhabdus, Blautia and Roseburia	Lv et al. (2019)
PC	Hyperlipoidemia	Golden hamster	Relative abundance of Ruminococcus, Oscillibacter, Bifidobacterium ↑	Tong et al. (2020)
BSGLP	Hyperlipidemia and fat accumulation	C57BL/6J mice	Allobaculum, Bifidobacterium, Christensenella-ceae-R-7 group and the abundance of Allobaculum, Bifidobacterium ↑ The abundance of Lachnospiraceae-UCG-001 and Rumini-clostrdium ↓	Sang et al. (2021)
GLP	T2DM	SD rats	The level of Blautia, Dehalobacterium, Parabacteroides and Bacteroides↑ Aerococcus, Ruminococcus, Corynebactrium and Proteus ↓	Chen et al. (2020)
EGLS	T2DM	SD rats	Proteobacteria community ↓	Jiang et al. (2021b)
F31	T2DM	SPF Kunming mice	Bacteroidetes ↑ Firmicutes ↓	Shao et al. (2022)
PSG	T2DM	Wistar rats	Beneficial bacteria such as *Bifidobacterium* and *Coprococcus* ↑*Enterococcus, Leuconostoc* and other harmful bacteria ↓	Ding (2020)
GLP	Insulin resistance and low-grade chronic inflammation	C57BL/6J mice	Relative abundance of *Actinomyceae* and *Leuconostoc* ↑ Relative abundance of *Spirillum* and *Aikman* ↓	Yang (2019)
CPGS and RPGS	Effect on spleen lymphocyte activity	BALB/c mice	Related to immune regulation *Adlercreutzia*, *Prevotella* and the unclassified *Desulfovibrionaceae* ↑ The enrichment of Parabacteroides ↓	Lu et al. (2021)
GS	Cardiac dysfunction	SD rats	The relative abundance of *Firmicutes* and *Proteobacteria* ↑ The abundance of Actinobacteria and Tenericutes ↓	Liu et al. (2021)
Liz-H	Cachexia	C57BL/6 mice	The relative abundance of *Ruminococcaceae* and *Bacteroides* ↑	Wu et al. (2023)
UB-GLS and B-GLS	Regulating gut flora	Stimulation of growth of lactic acid bacteria *in vitro*	The relative abundance of Ruminococcaceae, Bidobacteriaceae and Lactobacillaceae ↑ The abundance of Enterobacteriaceae and Lachnospiraceae ↓	Yang et al. (2020)
UGLS-O and BGLS-O	Regulating gut flora	10 volunteers	The relative abundance of beneficial bacteria such as *Prevotella*, *Faecalibacterium*, *Bifidobacterium* and *Lactobacillus* ↑ Relative abundance of *Escherichia coli* ↓	Kai et al. (2020)
GLO	Regulating gut flora	Fermentation model of gut flora *in vitro*	The relative abundance of beneficial bacteria such as Lactobacillus, Bifidobacterium, Faecalibacterium, and Prevotella ↑ The relative abundance of harmful bacteria such as *Escherichia-Shigella* and *Dorea* ↓	Xia et al. (2022)
GA	ALD	SPF Kunming mice	The levels of Lactobacillus, Faecalibaculum, Romboutsia,and Bifidobacterium ↑ The Helicobacter level ↓	Cao et al. (2022)
GAA	ALD	SPF Kunming mice	The abundance of Aerococcus, Bilophila and Bifidobacterium ↑ The proportion of Erysipelatoclostridium ↓	Lv et al. (2022)
GLE	ALD	SPF Kunming mice	The relative abundance of Ruminiclostridium-9, Prevotellaceae-UCG-001, Oscillibacter, [Eubacterium]-xylanophilum group, norank-f-Clostridiates-vadinBB60 group, GCA-900066225, Bilophila, Ruminococcaceae-UCG-009, norank-f-Desulfovibrionaceae and Hydrogenoanaerobacterium ↑ The proportion of *stricto-1* ↓	Guo et al. (2022)
WGL and AWGL	Intestinal mucosal epithelial malignant colon cancer	SD rats	The numbers of *Clostridium coccoides* and *Clostridium leptum* (secondary bile acids-producing bacteria) ↓	Yang et al. (2017)
GL55	Hyperlipoidemia	Wistar rats	*Alloprevotella, Ruminococcus* and *Prevotella* bacteria negatively correlated with the lipid profile ↑ The ratio of Turicibacter and Clostridium XVIII ↓	Rongkang et al. (2018)
GL95	Hyperlipoidemia	Wistar rats	Consistent with GL55	Guo et al. (2018)
GP	Hyperlipoidemia	Wistar rats	Alloprevotella bacteria ↑ The proportion of *Blautia* ↓	Tong et al. (2023)
GA	Hyperlipoidemia	SPF Kunming mice	Regulate *Barnesiella intestinihominis*, Bacteroides plebeins, *Akkermansia muciniphila*, *Lactobacillus murinus*, Parabacteroides goldesteinii, *Anaerotruncus colihominis*, *Barnesiella viscericola* and *Barnesiella intestinihominis* were negatively correlated with the caecal levels of SCFAs (acetic acid, butyric acid and propionic acid)	Guo et al. (2020)
GLFL	Effect on vascular risk factors	8 volunteers	Probiotics (such as *lactobacillus*); The number of *Firmicutes/Bacteroides* and conditional pathogenic bacteria ↑ The pathogens containing *Campylobacter* and *Aggregatibacter*; The number of *Lactococcus* belonging to probiotics ↓	Qizheng et al. (2017)
GLAA	Effect on sleep	ICR mice	*Bifdobacterium*, *Bifdobacterium animalis*, indole-3-carboxylic acid and acetylphosphate were negatively correlated with sleep latency, but positively correlated with sleep time and the hypothalamus 5-hydroxytryptamine concentration	Yao et al. (2021)
GLE+ Ciprofloxacin	Intestinal salmonella infection and side effects of ciprofloxacin drug treatment	C57BL/6J mice	The abundance of probiotics Lachnospiraceae NK4A136, Ruminococcaceae UGG-014, Lactobacillus, and Parabacteroides ↑	Li et al. (2023)
GLSO	Regulating immune function	ICR mice	The abundance of (Lactobacillus, Turicibacter and Romboutsia) and species (*Lactobacillus intestinalis* and *Lactobacillus reuteri*) ↑ Level of Staphylococcus and Helicobacter ↓	Wu et al. (2020)
SGP	Breast cancer and the side effects of PTX drug therapy	BALB/c mice	Five genera, such as *Bacteroides* and *Ruminococcus* ↑ The contents of Desulfovibrio and Odoribacter ↓	Su et al. (2018)
Gl	High cholesterol	C57BL/6 mice	The levels of Lactobacilla ceae family and Lactobacillus ↑	María et al. (2016)
GLPH	Hyperlipoidemia	Wistar rats	The contents of *Alistair* and *Clostridium* ↓	Xiong et al. (2022)
GAP and GAE	Colonitis	C57BL/6 mice	The abundance of Escherichia-Shigella, Bacteroides and Staphylococcus ↓	Li et al. (2021)
GLE	Colonitis	C57BL/6 mice	The abundance of Helicobacter and Bifidobacterium ↑	Li et al. (2022)
GLFB	Effect on immunosuppression	BALB/c mice	Gut flora imbalance was significantly improved by *Lactobacillus acidophilus* and Brevibacterium brevis	Li et al. (2021)
FGL	Chromium-induced body dysfunction	SD rats	Probiotics (such as Lactobacillus, Bifidobacterium and Roseburia) ↑	Dai et al. (2022)

## 2 Gut flora

Gut flora are the normal microorganisms in the human intestine. According to their function in the human intestine, Jie et al. (2021) divided them into three categories: probiotics (approximately 25%), neutral bacteria (approximately 50%), and pathogenic bacteria (approximately 25%). The main probiotics include *Bifidobacterium* and *Lactobacillus* (Ventura et al., 2012). The main pathogens include *Staphylococcus aureus*, *Salmonella*, *Proteus*, *Clostridium*, *Enterobacter*, and *Enterococcus*. Neutral bacteria include *Escherichia coli*, *Lactobacillus*, and *Streptococcus* (also known as fecal cocci). Studies have shown that the increase of the abundance of *Prevotella* can improve the function of glucose metabolism and enhance the storage capacity of glycogen (Kovatcheva-Datchary et al., 2015). *Faecalibacterium* can produce butyric acid and CO_2_ in colon, which has potential benefits for inflammatory bowel disease (IBD) and obesity suppression (La Fata et al., 2017; Healey et al., 2018) *Bifidobacterium* and *Lactobacillus* are typical anaerobic probiotics in intestinal bacteria. Probiotics represented by Bifidobacterium and lactobacillus can reduce cholesterol, resist tumor, delay aging, inhibit the adhesion of pathogenic bacteria, and relaxing bowel (Yang et al., 2013).

Gut flora has many functions such as developing immune system (Daniel and Ramnik, 2023), producing vitamins, maintaining intestinal cells and neutralizing toxins (Liu et al., 2022). Human health is dependent on intestinal microorganisms (Hongtao, 2022). The gut flora regulates intestinal homeostasis through interactions with the host (Xinyi, 2022), Because the ability of gut microbiota (GM) to produce short-chain fatty acids (SCFAs) is related to the host’s metabolic characteristics, which is a causal relationship between SCFAs and improvement of metabolic abnormalities (Tian et al., 2023). Functional components from natural products are widely used to treat a variety of intractable intestinal disorders due to their generally accepted safety profile in the treatment of diseases and efficacy with a long tradition of use (Wang et al., 2023). Gut flora imbalance will reduce beneficial substances, including SCFAs. The characteristics of an imbalanced or diseased gut flora are the decrease in beneficial bacteria and an enrichment of proteolytic flora, which will destroying the intestinal barrier and leading to a disruption in intestinal homeostasis. Gut flora imbalance can cause a wide range of chronic inflammatory diseases, including pancreatitis, enteritis, diabetes, asthma, atherosclerosis, and thrombosis (Sachdev and Pimentel, 2013). Gut flora usually regulates the human intestine by releasing different metabolites. In addition, the gut flora is easily influenced by external factors such as diet and living habits, and this can lead to metabolic diseases such as cardiovascular system (Jie et al., 2017) and digestive system (Li et al., 2017). As mentioned above, *G. lucidum* is rich in active ingredients such as polysaccharides and triterpenoids, which can be absorbed by the gastrointestinal tract, while macromolecules (polysaccharides) cannot be absorbed by the gastrointestinal tract, so it needs to be decomposed into micromolecules and then absorbed by the intestinal and hepatic circulation to play a pharmacological role.

## 3 Effects of different components of *Ganoderma lucidum* on gut flora in related diseases

### 3.1 *Ganoderma lucidum* polysaccharide (GLP)

GLP is one of the main active ingredients of *G. lucidum* and has many properties, including antibacterial, anti-inflammatory, anticancer, antioxidant, and antidiabetic functions (Liu et al., 2023). In addition, GLP can protect cardiovascular health, enhance the immune system (Guo et al., 2022), nourish and protect the liver (Faruque et al., 2023), and slow the progression of age-related health conditions (Wang et al., 2017). It has been reported that *G. lucidum* polysaccharides with different molecular weights have different protective effects on ethanol-induced acute gastric injury in rats, which proves that GLP can be used as a potential raw material for functional foods and dietary supplements (Tian et al., 2022). But so far, there is no report to summarize the mechanism of GLP in regulating the gut flora of diseases. Therefore, in this section, we will summarize the regulation mechanism of GLP on gut flora in different parts and ways from different angles of three lines of defense.

The intestinal immune system is the first line of defense against external pathogens. Extensive epidemiological investigations have shown that gut floral imbalances lead to chronic inflammation and diseases, such as chronic pancreatitis (CP), IBD, and colorectal cancer (CRC) (O’Keefe, 2016; Wong and Yu, 2019). Recently, although the pharmacological mechanism of food has been widely concerned by researchers, GLP stands out among many foods and has been recognized by many researchers. Jin et al. (2017) isolated and purified GLP from *G. lucidum* myceliumincreased the richness of microbiota in the cecum of Sprague–Dawley (SD) rats, and the ratio of *Firmicutes* and *Bacteroides* in rats in the GLP group was significantly reduced compared with the control group, proving that GLP can serve as a functional factor to regulate the intestinal barrier function. Li et al. (2016) induced ICR mice with diethyldithiocarbamate (DDC) and revealed the potential role of *G. lucidum* mycelium strain S3 (GLPs3) in the treatment of chronic pancreatitis (CP) mechanism. GLPs3 increased the relative abundance of probiotics and *Firmicutes* in mice, decreased the relative abundance of *Bacteroides*, significantly inhibited the inflammatory response of mice and made their intestinal barrier and gut flora tend to be healthy. Therefore, GLP can be used as a natural medicine to treat and prevent intestinal diseases, and provide data support for further study on the regulation of GLP on IBD gut flora in host immune system. Ulcerative colitis (UC) is one of the main types of IBD with chronic recurrent bowel disease. Xie et al. (2019) used edible *G. lucidum* polysaccharide GLP rich in β-glucan (>90%) to significantly alleviate diarrhea symptoms induced by dextran sulfate sodium (DSS) in Wistar UC rats because GLP The β-glucan in it can break down dietary polysaccharides and promote the growth of lactococci. Metagenomics and transcriptomic analysis revealed that GLP reduced the number of pathogens associated with acute diarrhea and increased the number of *Ruminococcus-1* and SCFAs. In addition, SCFAs produced by gut flora fermentation can regulate immune response, alleviate IBD and reduce the risk of colorectal cancer (CRC) (Llewellyn et al., 2018). Two groups of researchers conducted animal experiments on CRC induced by azomethane (AOM)/DSS and found that a variety of intestinal commensal bacteria and their metabolites help promote the occurrence of CRC (Sears and Garrett, 2014), they also found that gut flora imbalance (reduced microbial diversity) is one of the important factors leading to CRC (Dokht et al., 2022). Luo et al. (2018) extracting *G. lucidum* polysaccharides (GLPs) whose main component is β-1,3-glucan effectively prevents mice colon shortening and significantly reduces the relative abundance (RA) of cecal *Oscillospira*; Guo et al. (2021) water-soluble GLP extracted from peeled spores of *G. lucidum* reduced the relative abundance of some bacterial groups that are positively related to CRC carcinogenesis by inhibiting the TLR4/MyD88/NF-κB signaling pathway, and effectively improved the imbalance of gut flora in mice and increase the number of SCFAs. The above studies have shown that GLP can restore intestinal barrier function, reduce inflammatory responses and the risk of acquiring CRC, and enhance intestinal immunity, by reversing the flora that is positively related to the disease or increasing beneficial bacteria. However, the use of GLP as a supplement for patients with intestinal dysfunction or colitis requires further research to determine the most appropriate dosage for humans.

The liver is the largest detoxification organ in the human body and the second line of defense in the intestinal immune system. GLP can promote hepatocyte repair, prevent hepatic fibrosis, and also has pharmacological activity against metabolic diseases. Jiang et al. (2021a) *in vitro* fermentation of black *G. lucidum* polysaccharide (PSG) effectively improves the gut flora disorder in SD rats with acrylamide (AA)-induced liver injury, by increasing the relative abundance of *Ruminococcus* and *Lactobacillus* and reducing *Prevotella* It is a relative abundance, thereby achieving the liver protective effect. The edible *G. lucidum* spore powder (GLSP) produced by Leng et al. (2023) effectively improves alcoholic liver damage (ALD) induced by 50% ethanol. GLSP can not only reduce the levels of serum aspartate aminotransferase (AST) and alanine aminotransferase (ALT) increased by ALD, but also regulate aminotransferase to improve ALD and restore intestinal health. The animal experiment of GLP provides more guarantee for the pharmaceutical or clinical use of *G. lucidum*, and also provides treatment ideas for metabolic diseases such as long-term inflammation caused by obesity. Imran et al. (2018) extracted polysaccharides from *G. lucidum* fruiting bodies (GL-M) and mycelium (GL-F) for the treatment of high-fat diet (HFD)-induced obesity in mice. Polysaccharides significantly promote the growth of lactic acid-producing bacteria (LAP) that can improve the host digestive system and intestinal mucosa (Vigsnæs et al., 2013), changing the gut flora of obese mice by increasing the number of SCFAs and *Lactobacillus*. The GL-F group has more beneficial bacteria that can enhance metabolic capacity than GL-M. The enhanced metabolic capacity can achieve the effect of weight loss. Four groups of researchers have proved that different types of GLP and its compositions can improve the gut flora disorder caused by obesity, hyperlipidemia and fat accumulation to varying degrees. The water extract of *G. lucidum* mycelium (WEGL) of Chang et al. (2015) can inhibit obesity and improve the symptoms of low-grade chronic inflammation by improving the disorder of gut flora and maintaining the integrity of intestinal barrier in mice, it can alleviate dyslipidemia, inhibit obesity and steatosis, and improve the symptoms of low-grade chronic inflammation. Lv et al. (2019) *G. lucidum* polysaccharide peptide (GLPP) obtained from the fruiting body through alcohol extraction, precipitation, centrifugation, and freeze-drying can also reduce serum triglycerides (TG) and cholesterol (TC) in Wistar rats, free fatty acids (FFA) and low-density lipoprotein cholesterol (LDL-C) levels, inhibiting liver fat accumulation. Metagenomics analysis showed that the relative abundance of gut flora in high-fat rats was significantly changed, among which the number of *Bacteroidetes* was significantly increased and bile acid metabolism was accelerated, thereby improving lipid metabolism disorders. Tong et al. (2020) gave golden hamsters oral administration of a combination of *G. lucidum* fruiting body polysaccharide and chitosan (PC), which restored their lipid metabolism and gut flora disorders to normal. PC similar to GLPP, can reduce hyperlipidemia by reducing blood lipid content, and PC also additionally reduces AST content. In addition, PC also increased the relative abundance and SCFAs of beneficial bacteria negatively correlated with blood lipid profile. The (Sang et al., 2021) polysaccharide (BSGLP) extracted from the broken spores of *G. lucidum* significantly alleviates the upregulation of the TLR4/Myd88/NF-κB signaling pathway in mice adipose tissue and tends to reduce TG and non-esterified fatty acids (NEFA) levels, reversed the potential probiotics that were positively related to anti-obesity and improved hyperlipidemia. Ganoderma polysaccharide can regulate gut flora, which is partly responsible for inhibiting obesity, selectively improves the growth of benign bacteria, and improves metabolic syndrome by regulating gastrointestinal microbiota. I hope our summary can provide valuable data for the potential mechanism of GLP in treating dyslipidemia. Diabetes is a typical chronic metabolic disease in which type II diabetes mellitus (T2DM) accounts for more than 90% of patients who are diabetic (Ehtasham et al., 2022), especially those who are obese. Five groups of researchers obtained that different types of GLP can restore the gut flora of T2DM animals induced by HFD and STZ to normal level. Chen et al. (2020) and Jiang et al. (2021b) both used SD rat model. The former extracted and purified GLP from *G. lucidum* mycelium, which reversed the relative abundance of harmful bacteria such as *Ruminococcus* and *Proteus*, increased the number of beneficial bacteria such as *Bacteroides*, and finally made mice blood sugar returned to normal level. The latter made a grain fiber fraction which can not be digested in small intestine but can be fermented by microorganisms in colon. Resistant starch (RS) was used as coating material to prepare encapsulated *G. lucidum* spore polysaccharide (GLS), and the final product capsule was called EGLS. The intervention of EGLS significantly reduced the Proteus community and enhanced the parameters of glucose metabolism and fat metabolism in rats, which was related to enhancing insulin secretion, glycogen synthesis and reducing fat production. However, a kind of compound polysaccharide F31, which is rich in lactic acid bacteria, *Bacteroides* and *Ruminococeae*, isolated from the fruiting body of *G. lucidum* by Shao et al. (2022), can increase the specific pathogen-free (SPF) ratio of *Bacteroidetes/Firmicutes* (B/F) to 0.6969 (*p* < 0.01), even close to the normal control (*p* = 0.9579). Ding (2020) found that PSG could not be completely digested in rat’s stomach and small intestine, but was decomposed into SCFAs under the action of gut flora. PSG could significantly promote the growth of beneficial bacteria such as *Bifidobacterium* and fecal coccus, inhibit the growth of harmful bacteria such as *Enterococcus* and *Leuconostoc*, and change the index content related to T2DM, thus improving T2DM. Yang (2019) also found that GLP can improve low-grade chronic inflammation and reverse systemic insulin resistance induced by HFD in mice by reducing plasma insulin concentration and regulating inflammatory factors; Improve the gut flora structure of mice and enhance the sensitivity of mice to insulin resistance. To sum up, the hypoglycemic effect of GLP is mainly related to the gut flora that causes the increase of blood sugar. It can reduce blood sugar by promoting the increase of beneficial bacteria and the decrease of harmful bacteria, which has a positive effect on reducing inflammation. Therefore, GLP is an excellent potential candidate drug for preventing and treating T2DM.

Specific immunity is the third line of defense in the human body. The gut flora can affect the development and function of immune cells by regulating the functions of macrophages and T cells. In addition, the gut flora may participate in GLP-mediated immune regulation, but whether it plays a role in specific immune regulation needs further verification (Andrew et al., 2023). Lu et al. (2021) compared the changes in the activity of mice spleen lymphocytes between *G. lucidum* broken spore crude polysaccharide (CPGS) and *G. lucidum* broken spore polysaccharide (RPGS). CPGS and RPGS enriched *Adlercreutzia*, *Prevotella* and the unclassified *Desulfovibrionaceae*, which are positively related to immune regulation. The beta diversity of mice spleen lymphocytes in the RPGS group changed more obviously than that in the CPGS group. In addition, Liu et al. (2021) established a cardiac dysfunction model by intraperitoneal injection of trimethylamine-N-oxide (TMAO). *G. lucidum* spore extract (GS) can reduce the blood lipid content of TMAO rats and improve the index level of high density lipoprotein cholesterol (HDL-C) for preventing cardiovascular diseases. The lipophilic components rich in GS regulate the expression of related protein and polysaccharide components by targeting gut flora, so as to control the biotransformation of trimethylamine, thus maintaining the metabolic balance and function of the heart, which may be the potential mechanism of heart protection. Besides, *G. lucidum* polysaccharide (Liz-H) prepared by Wu et al. (2023) from malt extract agar (MEA) plate can treat cachexia induced by intraperitoneal injection of cisplatin combined with docetaxel (cisplatin+docetaxel), and Liz-H can restore disordered gut flora, reduce body weight and neutrophils in mice, and also alleviate muscle atrophy. It shows that Liz-H is a good chemical protective reagent. GLP plays a good role in promoting immune regulation. Although it provides data proof for *G. lucidum*’s natural functional food resources, it still needs more pharmacodynamics and clinical verification to establish the safety and feasibility of *G. lucidum*’s pharmacological action.

Up to now, according to the chemical structure of *G. lucidum* 1,3-or 1,6-b-d-glucan, GLP can not be digested by host-derived enzymes in human intestine, and there is no direct evidence that GLP can be absorbed by intestine. In view of the fact that there is no suitable polysaccharide digestive enzyme in human intestine, GLP can not be directly absorbed by human intestine. According to the research, we boldly speculate that GLP is likely to be decomposed by gut flora, and then regulate the changes of flora, and then play a therapeutic or auxiliary role in disease treatment.

### 3.2 *Ganoderma lucidum* oligosaccharides (GLOs)

Frontiers requires figures to be submitted individually, GLO is a chain-like homogeneous oligosaccharide extracted from *G. lucidum* polysaccharides, and its structure consists of a disaccharide repeating unit [−4-β-1-Galf(1–6)-O-(β-Glcp)-1-]_n_ = 3,4] (Isaac et al., 2013). GLO, a new functional sugar source, can be absorbed and utilized by beneficial gut flora. Because it cannot be degraded by gastric acid, GLO can play a role in regulating the gut flora (Wang et al., 2012).

Up to now, three groups of studies have confirmed that GLO can regulate different gut flora through *in vitro* fermentation model. Yang et al. (2020) found that oligosaccharides (UB-GLS and B-GLS) in broken and unbroken *G. lucidum* spore powder can increase the number of beneficial bacteria such as *Bifidobacterium* and *Lactobacillus*, increase the content of SCFAs, and regulate the function of gut flora. Kai et al. (2020) two kinds of oligosaccharides (UGLS-O and BGLS-O) prepared from (crushed and unbroken) *G. lucidum* spore powder by water extraction and alcohol precipitation can increase the relative abundance of beneficial bacteria such as *Prevotella*, *Faecalibacterium*, *Bifidobacterium* and *Lactobacillus*, but decrease the relative abundance of *Escherichia-Shigella*. In addition, Xia et al. (2022) extracted GLO from *G. lucidum* by ultrasonic-assisted enzymolysis and dextran gel electrophoresis, which can not only increase the beneficial bacteria detected by the first two groups of researchers, but also reduce the relative abundance of harmful bacteria such as *Escherichia-Shigella* and *Dorea.* Because GLO structure can not be digested and degraded in oral cavity, but can be used by gut flora in gastrointestinal tract, so GLO can be used as a component of functional food or nutritional products to improve intestinal tract and enhance immunity.

### 3.3 *Ganoderma lucidum* triterpenoids (GPs)

Ganoderma triterpene (GLT), also known as “ganoderic acid,” is one of the main active components isolated from *G. lucidum* (Baby et al., 2015). GLT compounds have high medicinal value and are widely used in cancer treatment, liver protection, prevention and treatment of cardiovascular diseases, epilepsy relief, asthma relief, reducing blood sugar and blood lipid levels, inhibiting platelet aggregation, radioprotection, and other areas; Compared to other active substances, GLP and GLT possess unique pharmacological advantages of more diverse and modifiable structures (Wei et al., 2019).

Diseases of the digestive system affect eating and nourishment. Gut flora affects the energy balance and weight of individuals by affecting the digestion and absorption of food, which has an important influence on the digestion process. Three groups of researchers found that different types of GLT can treat gut flora disordered by ALD, including main triterpenoid ganoderic acid (GA) (Cao et al., 2022), ganoderic acid A (GAA) (Lv et al., 2022) and *G. lucidum* ethanol extract (GLE) rich in ganoderic acid (Guo et al., 2022) significantly inhibited the abnormal elevated levels of serum TC, TG, LDL-C, AST and ALT, increased the level of serum HDL-C, and promoted the digestion and decomposition of alcohol by regulating different gut flora, thus protecting the liver from excessive accumulation of liver lipids and pathological changes caused by alcohol. The data show that alcohol-induced liver oxidative stress can be significantly improved by diet. Another common digestive system disease IBD is characterized by intestinal mucosal inflammation (Rodríguez-Lago et al., 2018). Yang et al. (2017) using 5% water extract of *G. lucidum* (WGL) and self-digested water extract of *G. lucidum* (AWGL) to treat intestinal mucosal epithelial malignant colon cancer achieved good results, which helped these extracts to restore normal colon mucosa by regulating gut flora and greatly reduced the number of cancer cells. However, AWGL significantly increased the level of propionate, which was more beneficial than WGL.

The management of metabolic diseases focuses on a light diet, and *G. lucidum*, with low fat and high cellulose content, is the first choice for many people. GLT can effectively treat three diseases (hypertension, hyperglycemia and hyperlipidemia) caused by metabolic disorder by regulating specific gut flora, lipid and cholesterol metabolites. Rongkang et al. (2018) found that supplementing 55% ethanol extract of *G. lucidum* (GL55) can increase the relative abundance of beneficial bacteria such as *Ruminococcus* beneficial to the metabolic process of gut flora in rats, and reduce the relative abundance of harmful bacteria such as *Fusobacterium*. Guo et al. (2018) using 95% ethanol to extract ganoderic acid (GL95) to supplement GL95 can improve the symptoms of hyperlipidemia, indicating that triterpenoids from *G. lucidum* can be used as a new functional food for potential treatment or improvement of hyperlipidemia. GL55, GL95 and GP (Tong et al., 2023) can all reduce serum TG, TC and LDL-C, and GL55 can additionally reduce ALT, FFA and fasting blood glucose levels to inhibit hepatic steatosis. However, GA extracted by Guo et al. (2020) regulates the changes of gut flora, which is different from the first three groups. *Barnesiella intestinihominis, Bacteroides plebeins, Akkermansia muciniphila, Lactobacillus murinus, Parabacteroides goldesteinii, Anaerotruncus colihominis, Barnesiella viscericola*, and *Barnesiella intestinihominis* were negatively correlated with the caecal levels of SCFAs (acetic acid, butyric acid and propionic acid), and increase the level of SCFAs in the intestine. To sum up, GLT will affect the gut flora, promote the increase of beneficial bacteria, reduce the number of harmful bacteria, regulate the serum level related to lipid metabolism and liver biochemical indicators, and maintain the intestinal environmental balance. We can realize the role of key microbial system types and important metabolic biomarkers in the occurrence and development of hyperlipidemia, and provide useful information for mining GLT drugs to prevent or treat hyperlipidemia. According to the existing research, we boldly speculate that gut flora may be a potential target for the treatment of dyslipidemia and nonalcoholic fatty liver disease (NAFLD), and GLT can regulate this target to achieve the purpose of treating diseases. We are looking forward to the follow-up research to confirm this speculation.

Peace of mind is important in the management of cardiovascular diseases, and *G. lucidum* nourishes the heart and calms the nerves. Qizheng et al. (2017) used *G. lucidum* mycelium fermentation broth (GLFL) to relieve cardiovascular diseases of volunteers to varying degrees and provide protection for human health; However, GLFL decreased the LDL-c of volunteers, increased the number of *Firmicutes/Bacteroides* and conditional pathogenic bacteria, and decreased the number of *Lactococcus* belonging to probiotics, which was unfavorable to human body. Therefore, GLFL as a drug needs more rigorous experiments to confirm the dose and quality. We call for re-examination of the dose and results of *G. lucidum* and its active ingredients in volunteer experiments, which may be different from rats. In addition, the sedative effect of *G. lucidum* has also been proved by Yao et al. (2021) to regulate gut flora and promote sleep. Taoist bacteria and metabolites rich in ethanol acidic fraction extract (GLAA) from *G. lucidum* mycelium, including *Bifdobacterium*, *Bifdobacterium animalis*, indole-3-carboxylic acid and acetylphosphate were negatively correlated with sleep latency, but positively correlated with sleep time and the hypothalamus 5-hydroxytryptamine concentration. GLAA administration decreased the levels of serum lipopolysaccharide and peptidoglycan in mice. We predict that GLAA can promote sleep in mice by relying on gut flora and serotonin-related pathways, and there is still much room for discussion on the relationship between gut flora and sleep.

Immune system is the fundamental way to cure diseases, and gut flora also has an important influence on the development and function of immune system. *G. lucidum* ethanol extract (GLE) (Li et al., 2023) is beneficial to gut flora and immune system. Ordinary ciprofloxacin can destroy the intestinal barrier and increase the risk of pathogenic bacterial infection in mice. GLE+ ciprofloxacin combined administration can avoid the intestinal barrier by increasing the abundance of probiotics *Lachnospiraceae* NK4A136, *Ruminococcaceae* UGG-014, *Lactobacillus*, and *Parabacteroides*. It shows that GLE has great potential in repairing intestinal injury caused by antibiotics. Wu et al. (2020) used supercritical carbon dioxide fluid extraction technology to extract oily lipids from broken *G. lucidum* spores. *G. lucidum* spore oil (GLSO) rich in triterpenes and ergosterol can regulate gut flora, enhance phagocytosis of macrophages and cytotoxicity of NK cells, and improve immunity. The above research shows that gut flora has an important influence on the function of immune system. GLT can alleviate the side effects caused by other drug treatments and enhance immunity by regulating gut flora, which is beneficial to physical and mental health.

### 3.4 Combined application of active components of *Ganoderma lucidum* and clinical drugs

Su et al. (2018) from the perspective of the role of gut flora in tumor, a mice model of breast cancer was established. Through 16S rRNA sequencing, it was found that *G. lucidum* spore polysaccharide (SGP) could improve the gut flora imbalance caused by paclitaxel (PTX). It was found that the bacterial abundance of five genera, such as *Bacteroides* and *Ruminococcus*, increased significantly, while the abundance of the genera of *Acinetobacte*r and *Vibrio desulphulariae*, which increased the risk of tumor, decreased.

Obesity is the result of nutritional metabolic disorder, which leads to slow cholesterol metabolism and increased TC concentration in the blood of obese patients, thus leading to high cholesterol levels. María et al. (2016) firstly treated hypercholesterolemia with ethanol-water extract (Gl) from *G. lucidum* mycelium from Mexico, and compared the advantages and disadvantages with simvastatin. The effect of Gl extract on lipid metabolism was better than simvastatin. The levels of serum TG, TC, LDL-C, liver cholesterol and liver triglyceride in Gl group are closer to the normal level than simvastatin. The decrease of TC level is mediated by α-glucan and β-glucan in Gl, which promotes the absorption of cholesterol in intestinal tract and increases the levels of *Lactobacilla ceae family* and *Lactobacillus*. Secondly, Xiong et al. (2022) can also treat hyperlipidemia with *G. lucidum* protease hydrolysate (GLPH), and reduce the content of *Alistair* and *Clostridium* in rat intestine, and improve the disorder of lipid metabolism. *G. lucidum* polysaccharide extract (GAP), *G. lucidum* 75% ethanol extract (GAE) (2021) and *G. lucidum* ethanol extract triterpenoid (GLE) (2022) of Li et al. all promoted the recovery of colitis in a manner dependent on gut flora. GLE also increased the abundance of *Helicobacter* and *Bifidobacterium*, enhanced immunity and protected intestinal barrier. It can be seen that the above experiments reveal that *G. lucidum* has a significant positive regulatory effect on gut flora in metabolic diseases, indicating that *G. lucidum* has the potential as a lipid-lowering functional food beneficial to metabolism.

*Ganoderma lucidum* not only promotes body metabolism, but also has a positive effect on body function recovery. Two groups of researchers conducted *in vitro* fermentation experiments from the perspective of immune function of gut flora. Li et al. (2021) adding *Lactobacillus acidophilus* and *Brevibacterium* to the fermentation broth of water extract of *G. lucidum* fruiting body restored the T lymphocyte activity of dexamethasone (DEX)-induced immunosuppression mice to normal level, and the gut flora imbalance was obviously improved. In contrast, Dai et al. (2022) used *G. lucidum* tablets (FGL) fermented by *Lactobacillus rhamnosus* to treat chromium-induced physical dysfunction in SD rats, increased the content of probiotics and stopped diarrhea in rats. These positive changes once again show that *G. lucidum* can enhance immunity by regulating gut flora and play an immunomodulatory role. The targeted regulation of *G. lucidum* or *G. lucidum* combination drugs on gut flora and its metabolites may be the key to enhance the immune function of the body, which provides a reliable basis for the application of probiotic fermented *G. lucidum* in immune regulation.

## 4 Conclusion and prospects

**Figure 3:**
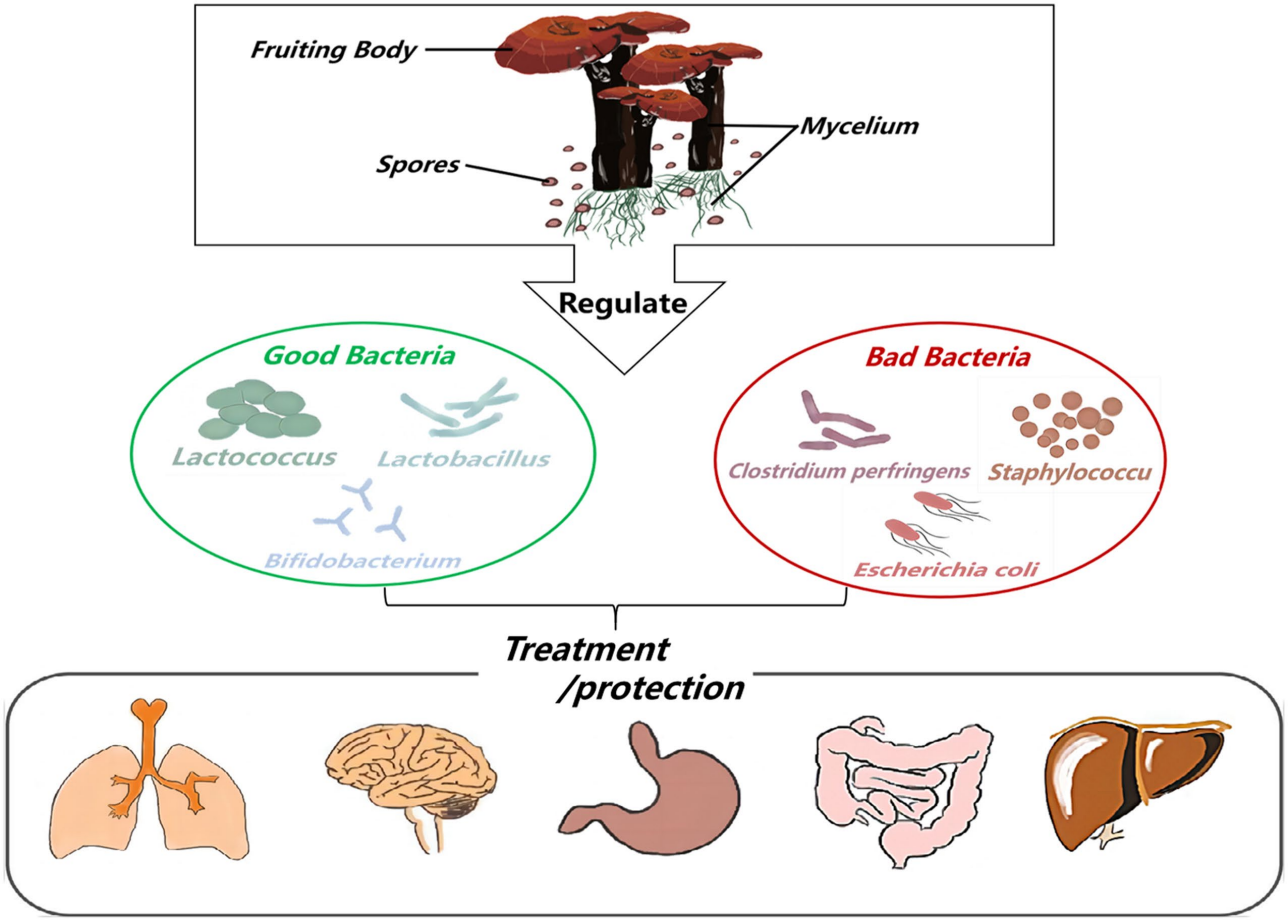
The flow chart of Ganoderma lucidum and its active ingredients for treating diseases or protecting the body by regulating gut flora.

*Ganoderma lucidum* possesses a variety of biological activities and has no obvious toxicity or side effects as a pharmaceutical ingredient. Because its consumption worldwide is estimated at several thousand tons, and the market is growing rapidly (Liu, 2021; Figure 3). This huge consumption potential and planting ability make *G. lucidum* can be used to develop high-quality functional drugs and foods in terms of composition, pharmacological action and clinical application (Lai et al., 2004; Fidelis et al., 2023). Therefore, pharmacists, medical scientists, and chemists have attached great importance to this issue. This paper reveals the relationship among the active components of *G. lucidum*-gut flora-diseases, and summarizes the effects of *G. lucidum* and its active components on improving related diseases by regulating gut flora. Animal and *in vitro* experiments show that when the body is infected by pathogenic bacteria or suffers from a disease, the intestinal microecological balance is destroyed, and active components such as *G. lucidum* polysaccharides and triterpenoids can participate in the body metabolism process from the perspective of regulating gut flora. Most of its manifestations involve increasing the relative abundance of probiotics (such as lactic acid bacteria and *Bifidobacteria*) and reducing the relative abundance of pathogenic bacteria (such as *Escherichia-Shigella* and *Cladosporium*) to maintain the dynamic balance of gut flora in terms of microbial species and abundance (Shuai, 2021). To provide scientific basis for clinical application of *G. lucidum*.

*Ganoderma lucidum* and its active components exert protective effects by regulating intestinal imbalance, preventing diabetes and nephropathy, and alleviating kidney damage caused by chemotherapy drugs. Basic and applied research on the active components of *G. lucidum* (He et al., 2023) will certainly promote its use as a medicine or health food in the prevention and treatment of diseases in terms of reasonable diet (Gentile and Weir, 2018), scientific medicine, and health care. Foreign researchers have found that *G. lucidum* can inhibit intestinal inflammation and relieve symptoms such as intestinal sensitivity. The research on *G. lucidum* and gut flora in China is also deepening. *G. lucidum* has antibacterial effect on some pathogens in the intestine, which is helpful to prevent and treat intestinal infections. In a word, the regulating and improving effect of *G. lucidum* on gut flora has been widely concerned and studied. In the future, with the deepening of research and technological progress, we are expected to have a deeper understanding of the interaction mechanism between *G. lucidum* and gut flora and provide more effective solutions for intestinal health.

Although *G. lucidum* has been actively developed in the intestinal field, we should also pay attention to the objective problems that cannot be ignored in its development (El Sheikha, 2022). First, food safety problems in the environment are serious, and *G. lucidum* is affected by environmental pollution. When extracting *G. lucidum* components, heavy metal ions are inevitably present; their cytotoxicity must be avoided, and food safety must be guaranteed. Second, there is the problem of purity. There are many active ingredients in *G. lucidum*, but not all of them are beneficial; therefore, innovations in separation and purification technology are needed. Finally, there is the issue of norms. Extensive processing of *G. lucidum* has high economic benefits, but there are bound to be problems arising from variations in techniques and counterfeiting. However, from the perspective of food safety and standardization, *G. lucidum* can be developed to produce cost-effective products for regulating gut flora, improving health care, and enhancing recovery from illness.

## Author contributions

XQ: Conceptualization, Writing – original draft, Writing – review & editing. ZF: Data curation, Investigation, Writing – original draft. JZ: Data curation, Writing – review & editing. WZ: Visualization, Writing – review & editing. NZ: Formal analysis, Methodology, Writing – original draft. XW: Conceptualization, Funding acquisition, Resources, Supervision, Writing – original draft, Writing – review & editing.
